# Natural Course and Long-Term Outcomes of Gastric Subepithelial Lesions: A Systematic Review

**DOI:** 10.3390/jcm14041055

**Published:** 2025-02-07

**Authors:** Masaya Iwamuro, Hiroyuki Okada, Motoyuki Otsuka

**Affiliations:** 1 Department of Gastroenterology and Hepatology, Okayama University Graduate School of Medicine, Dentistry and Pharmaceutical Sciences, Okayama 700-8558, Japan; 2 Department of Internal Medicine, Japanese Red Cross Society Himeji Hospital, Hyogo 670-8540, Japan

**Keywords:** esophagogastroduodenoscopy, gastric lesions, gastrointestinal stromal tumor, subepithelial lesion, submucosal tumor

## Abstract

**Background/Objectives:** Gastric subepithelial lesions (SELs) are often incidentally detected during endoscopic examinations, with most patients being asymptomatic and lesions measuring <20 mm. Despite their generally indolent nature, certain SELs, such as gastrointestinal stromal tumors, require resection. Current guidelines recommend periodic surveillance; however, the natural course and long-term outcomes of gastric SELs have not been sufficiently investigated. This systematic review aimed to synthesize evidence on the progression, growth rate, and risk factors associated with gastric SELs to inform clinical management strategies. **Methods:** A comprehensive search of PubMed was conducted for peer-reviewed studies published between January 2000 and November 2024. Eligible studies included original studies on the follow-up and progression of gastric SELs. Non-English articles, reviews, case reports, and unrelated topics were excluded. In total, 277 articles were screened, with 15 additional articles identified through manual screening. Ultimately, 41 articles were included in the analysis. The study protocol is registered in PROSPERO (CRD42024614865). **Results:** Large-scale studies reported low growth rates of 2.0–8.5% over 2.0–5.0 years, while smaller studies reported a broader range of growth rates of 5.4–28.4%. The factors contributing to these discrepancies include patient selection, follow-up duration, and growth criteria. Risk factors for lesion size increase include larger initial lesion size, irregular margins, heterogeneous echo patterns, and certain tumor locations. **Conclusions:** These findings underscore the need for individualized management strategies based on lesion size, imaging characteristics, and risk factors. The close monitoring of high-risk lesions is crucial for timely intervention. Standardized growth criteria and optimized follow-up protocols are essential for improving clinical decision making and patient outcomes.

## 1. Introduction

**Figure 1:**
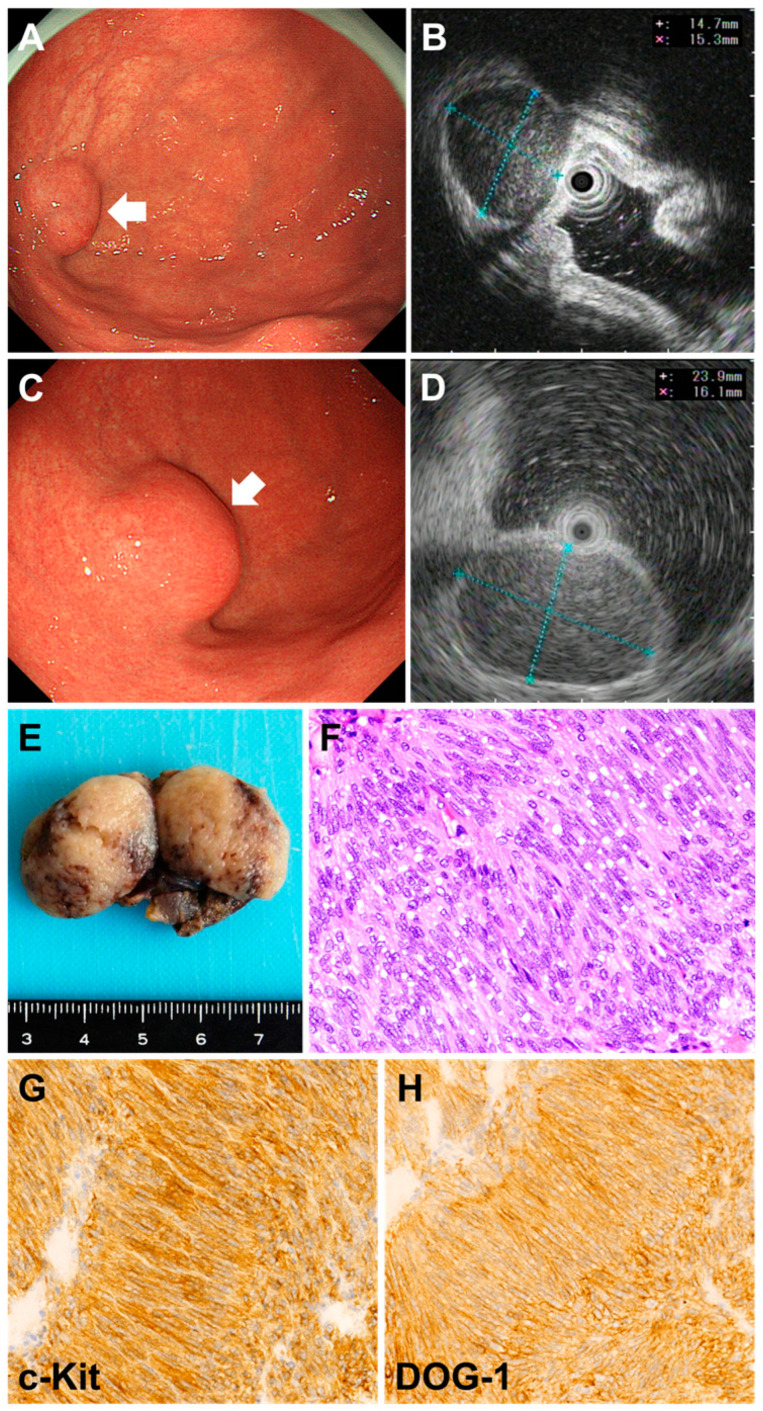
Images of a case of a gastrointestinal stromal tumor (GIST). A 20 mm subepithelial lesion is observed in the gastric fornix (**A**, arrow). Initial endoscopic ultrasonography (EUS) revealed a 15 × 15 mm spherical mass, a hypoechoic tumor continuous with the muscularis propria, and slightly heterogeneous internal echoes (**B**). After 21 months, EUS revealed that the lesion had grown to 24 × 16 mm (**C**,**D**). EUS-guided fine-needle aspiration biopsy diagnosed the lesion as a GIST, and the tumor was resected (**E**). Histologically, the spindle-shaped tumor cells show dense fascicular and palisading proliferation (**F**, hematoxylin and eosin staining, ×20). Immunostaining was positive for c-Kit (**G**, ×20) and DOG-1 (**H**, ×20) and negative for S-100, leading to a final diagnosis of GIST. The mitotic count was ≤5/50 high-power fields.

Most gastric subepithelial lesions (SELs) identified during endoscopic examinations are small, typically measuring less than 20 mm in diameter [1,2,3,4,5,6]. These lesions are usually asymptomatic, have no noticeable clinical signs, and are most often incidentally detected during examinations performed for unrelated reasons. Most gastric SELs belong to the category of mesenchymal tumors, which includes gastrointestinal stromal tumors (GISTs; Figure 1), myogenic tumors, such as leiomyomas and leiomyosarcomas, and neurogenic tumors, such as schwannomas. GISTs and leiomyomas are the most frequently encountered types of tumors in the stomach, accounting for a significant proportion of cases.

In addition to neoplastic growths, gastric SELs can arise from various non-neoplastic conditions (Table 1). These non-neoplastic lesions include heterotopia in the stomach, such as an ectopic pancreas and heterotopic spleen, which can mimic neoplastic lesions on imaging and endoscopy and often pose diagnostic challenges. Vascular anomalies, including gastric varices and extramural compression by aneurysms, are additional causes of subepithelial-like lesions that can be mistaken for tumors. Furthermore, submucosal cystic formations, such as duplication cysts and extrinsic compressions arising from structures outside the gastric wall, including adjacent organs, masses, or liver cysts, may contribute to the diverse spectrum of SELs observed during endoscopy. While this broad range of conditions collectively falls under the umbrella term ‘gastric SELs’ and should always be considered in differential diagnosis, they typically lack malignant potential and are rarely associated with significant growth during long-term follow-up, distinguishing them from their neoplastic counterparts.

Detailed analyses of the composition of gastric SELs are limited. This is primarily due to the fact that most SELs are not subjected to histopathological evaluation, making it challenging to accurately determine the proportion of each lesion type. In one study involving 169 cases of gastric SELs in which pathological diagnoses were confirmed or histological features were inferred based on endoscopic ultrasound (EUS) findings, GISTs were identified as the most common (39.1%), followed by ectopic pancreas (22.5%), leiomyomas (18.3%), lipomas (6.5%), non-GIST malignant tumors (1.2%), and neuroendocrine tumors (0.6%) [7].

Although current clinical guidelines generally advocate for periodic surveillance as an appropriate management approach for most gastric SELs [1,2,3,4], particularly smaller and asymptomatic ones, the natural history and long-term behavior of these lesions remain inadequately understood. To address this knowledge gap, we conducted a systematic review of published studies that have specifically explored the natural course and progression of gastric SELs.

## 2. Methods

We performed a comprehensive search of PubMed to identify peer-reviewed articles published between 1 January 2000 and 10 November 2024 without imposing any restrictions on study design. To supplement this, we manually screened reference lists of the selected articles that fulfilled the eligibility criteria for additional relevant studies. The search, conducted by the lead author (M.I.), used the following keywords: “subepithelial lesion” or “submucosal tumor” in combination with “course” and “stomach” or “gastric”. Articles were considered eligible for inclusion if they involved follow-up studies on the progression of gastric SELs and presented original studies. Studies were excluded if they were written in languages other than English; if they were categorized as review articles, case reports, or case series; if they focused on animal- or cell-based studies; or if they addressed topics unrelated to the follow-up of gastric SELs. Full texts of all eligible articles were reviewed and analyzed in detail.

This systematic review was conducted in accordance with the Preferred Reporting Items for Systematic Reviews and Meta-Analyses (PRISMA) guidelines. Although we initially intended to evaluate the risk of bias for all included studies using the Newcastle–Ottawa Scale, no cohort or case–control studies were identified in our search results; only single-arm observational studies were available. Consequently, the risk of bias for the included single-arm observational studies was qualitatively assessed by two independent reviewers (M.I. and H.O.) based on the following criteria: the methodological quality of the studies, with a focus on the clarity of study objectives, inclusion and exclusion criteria, methods of outcome measurement, and potential sources of bias, including selection and information bias.

This study protocol was prospectively registered with PROSPERO to ensure transparency and reduce bias. The registration number for this study was PROSPERO 2024 (CRD42024614865).

## 3. Results

### 3.1. Search Results

**Figure 2:**
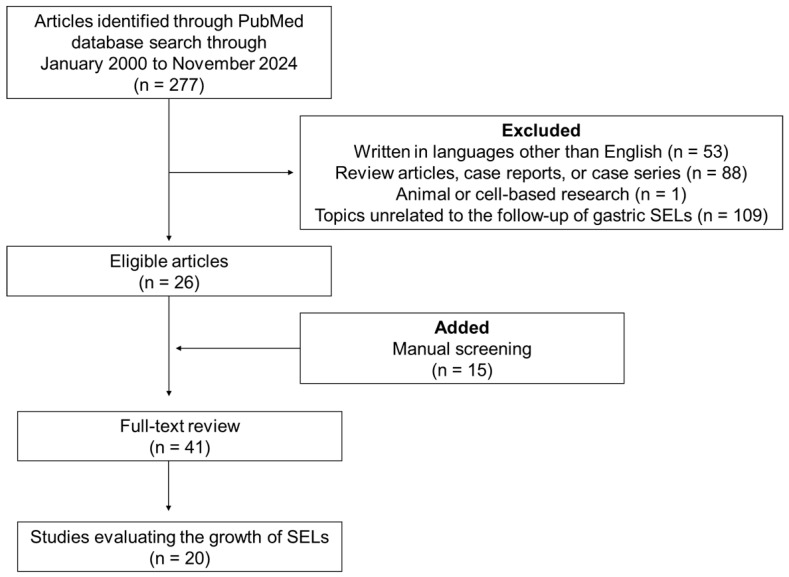
Flow diagram detailing the process of identifying, screening, assessing eligibility, and excluding studies in the literature search. SELs: subepithelial lesions.

Figure 2 provides a flow diagram detailing the process of identifying, screening, assessing eligibility, and excluding studies from the literature search. An initial search yielded 277 articles. Of these, 53 were excluded because they were written in languages other than English; 88 were review articles, case reports, or case series; 1 focused on animal- or cell-based studies; and 109 addressed topics unrelated to the follow-up of gastric SELs. After applying the exclusion criteria, 26 articles were selected from the initial PubMed search. An additional 15 articles were included after a manual review of the relevant studies. Forty-one articles were subjected to full-text review. Finally, 20 articles provided relevant findings regarding the follow-up of gastric SELs [7,8,9,10,11,12,13,14,15,16,17,18,19,20,21,22,23,24,25,26].

### 3.2. Growth Rates of Gastric SELs During Follow-Up

As shown in Table 2, a review of the available literature on the natural course of gastric SELs revealed a clear discrepancy in reported growth rates between large- and small-scale studies. Studies involving >410 patients consistently reported relatively low growth rates of 2.0–8.5% over follow-up periods ranging from 2.0 to 5.0 years [16,19,23,24,26]. In contrast, studies with smaller sample sizes (fewer than 145 patients) reported a wide range of growth rates, between 5.4% and 28.4%. These studies, with smaller sample sizes, exhibited bimodal growth rates. In two studies that used only esophagogastroduodenoscopy for follow-up, the growth rates were 5.4% and 6.3% [14,15]. In contrast, studies that tracked SELs using either EUS alone or a combination of EUS and esophagogastroduodenoscopy reported significantly higher growth rates ranging from 10.2% to 28.4%. Among the smaller studies, approximately half (7 out of 15) documented growth rates exceeding 20%. Several factors likely contributed to this discrepancy, including differences in lesion risk profiles, patient selection bias, follow-up duration, and growth assessment criteria.

One plausible explanation is that large-scale studies tend to include a broader and more general population, which often includes a higher proportion of low-risk lesions. These studies typically reflect real-world clinical settings, in which the majority of gastric SELs are small, asymptomatic, and benign. For example, in the studies by Song et al. [19], Abe et al. [23], and Iwamuro et al. [26], the diagnosis of SELs was based solely on endoscopic findings without EUS. Although this approach closely reflects routine clinical practice, the inclusion criteria may include non-tumorous lesions, such as vascular anomalies and extrinsic compressions, along with true solid tumors, such as GISTs and leiomyomas. Theoretically, non-tumorous lesions are less likely to exhibit significant growth or undergo malignant transformation during the study period, which may account for the lower reported overall growth rates. In contrast, small-scale studies are often more focused, with a tendency to include lesions that are already suspected or confirmed to be high-risk for size increase, such as GISTs. These studies may have selected cases based on prior EUS findings or histopathological diagnoses, thereby introducing an inherent bias toward lesions that are more likely to grow. For example, Sawaki et al. investigated diagnosed cases of GISTs and reported a growth rate of 11.1% [9], whereas Lachter et al. evaluated pathologically diagnosed GISTs using EUS and observed a growth rate of 20.0% [10]. Similarly, Hu et al. focused on 10–30 mm myogenic tumors and reported a growth rate of 28.4% [22]. This selective inclusion of high-risk lesions in smaller studies increases the reported growth rates and contrasts with the findings of larger, more representative studies.

The duration of follow-up may also have influenced the observed discrepancies in growth rates. Large-scale studies often report relatively short follow-up periods of approximately 2.0–5.0 years, which may not be sufficient to detect the slow growth of indolent lesions. In comparison, smaller studies frequently involved longer follow-up durations, during which even slow-growing lesions are more likely to show a measurable increase in size. Consequently, differences in follow-up timeframes between large and small studies may partially account for the higher growth rates observed in smaller studies.

Finally, the variability in the criteria used to define lesion growth contributed to the observed differences in outcomes. Large-scale studies often apply strict and objective thresholds for growth assessment, such as an increase of ≥5 mm, as seen in our study [26], which reported a growth rate of only 5.7%. Conversely, smaller studies may adopt more sensitive criteria, such as a ≥20% increase in size, as exemplified by the study of Hu et al. [22], which reported a growth rate of 28.4%. Differences in the evaluation modalities may also have significantly influenced the sensitivity of SEL growth detection. EUS allows the precise measurement of lesion sizes as small as 0.1 mm, enabling the detection of subtle changes. However, when esophagogastroduodenoscopy is used alone, lesion growth can only be assessed visually, making it challenging to identify minor size changes. Consequently, growth may only be observed when the lesion exhibits a marked increase in size. This distinction is reflected in the bimodal growth rates observed in studies with smaller sample sizes. Two studies that used only esophagogastroduodenoscopy for follow-up reported low growth rates (5.4% and 6.3%), whereas studies that tracked SELs using either EUS alone or a combination of EUS and esophagogastroduodenoscopy reported significantly higher growth rates (10.2–28.4%). The use of different growth definitions and modalities for tracking SELs inevitably affects the reported outcomes, and likely contributes to the wide range of growth rates observed in previous studies.

### 3.3. Doubling Time of Gastric SELs

The doubling time of gastric SELs is an important parameter for assessing tumor growth rates and potential malignancy. Various studies have reported a wide range of doubling times in gastric SELs. Koizumi et al. found that the doubling time for GISTs was 17.2 months, with intermediate- and high-risk GISTs having doubling times of <6 months [20]. Based on this finding, they recommended that initial follow-up examinations should be conducted within at least the first six months after diagnosis, even for small SELs <20 mm in diameter. Ueyama et al. suggested that tumors with a doubling time of <16 months should be considered suspicious for malignancy because such lesions tend to grow more rapidly [27]. This underscores the importance of doubling time as a critical indicator for monitoring tumor progression. Hata et al. reported a similar doubling time of 19.2 months for gastric gastrointestinal mesenchymal tumors [18]. In contrast, Shiratori et al. observed that 39 of 145 gastric mesenchymal tumors (26.9%) increased in size with a relatively long mean doubling time of 3.6 years [25].

### 3.4. Risk Factors for Size Increase in Gastric SELs

Subepithelial lesions of the stomach, particularly GISTs, exhibited varied growth patterns during follow-up, with some showing a significant increase in size over time. Identifying the factors that predict the likelihood of growth in SELs is critical for managing patient follow-up and treatment decisions. Several studies have investigated the risk factors associated with tumor progression, including tumor size, margin irregularities, and specific clinical and imaging features as significant contributors.

One of the most consistent risk factors for tumor growth is initial tumor size. Larger SELs have a higher probability of increasing in size during follow-up. Several studies have shown that tumors >20 mm are more likely to undergo size progression than smaller lesions [19,24,28,29]. In a study by Fang et al., tumors >14 mm were associated with a significantly higher rate of progression (35.7%) than those less than this threshold (2.8%) [17]. Similarly, other studies have reported that tumors >9.5 mm or 14 mm are more likely to show growth [24,25]. These thresholds vary across studies, emphasizing the necessity of determining a value that truly reflects the risk of tumor growth. An additional concern is that SELs smaller than these thresholds can grow over time, suggesting the importance of designing surveillance intervals tailored to the initial tumor size.

EUS findings are another important predictor of tumor progression. Tumors with irregular margins or extraluminal borders on EUS are more likely to increase in size. For example, Shiratori et al. found that GISTs with irregular extraluminal borders had a higher hazard ratio for growth (3.65), suggesting that such lesions are more prone to size increases [25]. Tumors with heterogeneous echo patterns or those exhibiting cystic spaces are also more likely to show progression, as these features are often associated with malignant or potentially malignant lesions [8,13,23]. Melzer et al. found that homogenous hypoechoic lesions had a lower chance of growth than those that transitioned to a non-homogenous pattern [8]. Additionally, calcification within a tumor is associated with a lower likelihood of growth [25].

The location of the lesion within the stomach may affect the growth potential. Tumors located in non-upper areas of the stomach, such as the body or antrum, may exhibit a higher risk of growth than those in the upper regions [18]. Our investigation of gastric leiomyomas, schwannomas, and GISTs revealed a higher likelihood of SELs around the gastric cardia being leiomyomas than schwannomas or GISTs [30]. This observation suggests a potentially lower likelihood of growth of SELs in the gastric cardia than in other regions of the stomach. However, in a prospective observational study of 560 gastric SELs, no significant differences in growth rates were observed among SELs located in the upper, middle, and lower thirds of the stomach [26]. Age has also been identified as a factor because older patients are more likely to experience tumor growth [18].

In summary, several factors contribute to the risk of growth of gastric SELs, particularly GISTs. Larger tumor size, irregular margins or extraluminal borders on EUS, heterogeneous echo patterns, and specific tumor locations are key indicators for monitoring potential growth. Identifying these risk factors allows clinicians to tailor follow-up strategies and prioritize the closer surveillance of patients with a higher likelihood of progression.

### 3.5. Limitations

This systematic review had several limitations. First, the included studies were predominantly single-arm observational studies lacking cohort or case–control designs, which limited our ability to assess causation and generalizability. Second, there was considerable heterogeneity among the included studies in terms of patient selection, follow-up duration, definition of lesion growth, and outcome measurement methods. In particular, the term ‘gastric SELs’ encompasses a wide range of heterogeneous entities that may have influenced the varying proportions of higher-risk lesions, such as GITSs, selected based on endoscopic ultrasonography findings. This review also included studies with relatively short follow-up periods. These heterogeneities may have contributed to the wide range of reported growth rates and risk factors. Third, publication bias cannot be ruled out, as only studies published in English were included, potentially excluding relevant findings from non-English publications. Additionally, the reliance on retrospective data in many studies raises concerns regarding selection and information biases. Finally, the lack of standardized growth criteria and uniform follow-up protocols across studies underscores the need for future research to address these methodological gaps and improve the reliability of findings in this field.

## 4. Conclusions

This systematic review highlights the diverse natural courses and long-term outcomes of gastric SELs and offers valuable insights into their growth potential and risk factors. Gastric SELs, particularly GISTs, display varying growth patterns depending on their size, location, and imaging characteristics. Large-scale studies, which include a broader population of low-risk lesions, report relatively low growth rates (2.0–8.5%), whereas small-scale studies focusing on high-risk lesions tracked with EUS show relatively higher growth rates (10.2–28.4%). These discrepancies underscore the importance of careful patient selection, longer follow-up duration, and consistent growth criteria when evaluating the natural history of gastric SELs.

The findings of this review highlight the importance of individualized management strategies tailored to the lesion characteristics and risk profiles. The close monitoring of high-risk lesions, particularly those with larger sizes or specific ultrasonographic features, is essential for timely intervention. Future studies should prioritize standardizing growth assessment criteria and refining follow-up and treatment protocols. Incorporating these insights into clinical practice will support better decision making and improve long-term outcomes in patients with gastric SELs.

## Figures and Tables

**Table 1:** Major subepithelial lesions observed in the stomach.

1. Mesenchymal Tumors:
Gastrointestinal stromal tumor (GIST);
Leiomyoma;
Leiomyosarcoma;
Schwannoma;
Glomus tumor.
2. Ectopic or Heterotopic Tissues:
Ectopic pancreas (heterotopic pancreas);
Heterotopic spleen (splenosis).
3. Cystic Lesions:
Duplication cyst;
Retention cyst, including submucosal cysts associated with *H. pylori* gastritis.
4. Vascular Lesions:
Hemangioma;
Lymphangioma.
5. Inflammatory or Reactive Lesions:
Inflammatory fibroid polyp (Vanek’s tumor);
Anisakiasis.
6. Neuroendocrine and Other Neoplasms:
Neuroendocrine tumor;
Granular cell tumor;
Lipoma;
Gastric cancer mimicking subepithelial tumor;
Lymphoma;
Metastatic tumor.
7. Others:
Varices (e.g., gastric varices);
Pseudo-lesions (e.g., submucosal hematoma, extramural compression).

**Table 2:** Summary of studies on the growth of gastric subepithelial lesions.

No.	Author	Year	No. Lesions	Study Design	Type of Lesions	Follow-Up Method	Follow-Up Period	Definition of Size Increase	Results
1	Melzer E	2000	16	NA	<40 mm SELs	EUS	16.7 months (mean)	Increase in size (≥25%)	25.0%
2	Sawaki A	2006	16	Retrospective	GISTs diagnosed by EUS-FNA	EUS and EGD	4.9 years (median)	Increase in size (≥20%)	11.1%
3	Lachter J	2008	70	Retrospective	Pathologically diagnosed GISTs	EUS	5.4 years (mean), 5 years (median)	Increase in size (>1 mm/month)	20.0%
4	Gill KR	2009	51	Retrospective	<30 mm SELs of second and fourth echo layer	EUS	29.7 months (mean)	Increase in size or change in echogenic features	13.7%
5	Bruno M	2009	49	Prospective	<30 mm SELs of fourth echo layer	EUS	31 months (mean)	Increase in size (≥25%)	10.2%
6	Lok KH	2009	23 *	Retrospective	SELs of fourth echo layer	EUS	17.3 months (mean)	Increase in size (≥5 mm)	13.0% *
7	Lim YJ	2010	130	Retrospective	SELs	EUS and EGD	59.1 mo (mean) *	Increase in size (≥ 5 mm and >25%)	5.4%
8	Chien CH	2010	9	Retrospective	GISTs suspected on EUS	EUS	23 months (mean)	Increase in size (>5 mm)	11.1%
			16	Retrospective	GISTs suspected on EUS	EGD	28 months (mean)	Increase in size (>5 mm)	6.3%
9	Kim MY	2011	989	Retrospective	≤30 mm SELs of second–fourth echo layer	EUS	24 months (median)	Increase in size, change in echogenicity, or morphology	8.5%
10	Fang YJ	2012	50	Retrospective	<30 mm SELs	EUS	39.2 months (mean)	Increase in size (≥20%)	28.0%
11	Hata S	2013	50	Retrospective	SELs of second or fourth echo layer	EUS	24.3 months (mean)	Increase in size (>3 mm)	28.0%
12	Song JH	2015	640	Retrospective	SELs	EGD	47.3 months (mean) *	Increase in size (≥25%)	4.2%
13	Koizumi S	2016	47	Retrospective	Pathologically diagnosed SELs	EUS	31.7 months (median) *	NA **	NA **
14	Gao Z	2017	69	Retrospective	≤20 mm SELs of second–fourth echo layer	EUS	28 months (mean)	Increase in size (≥20%)	23.2%
15	Hu ML	2017	88	Retrospective	10–30 mm myogenic tumors	EUS	26.3 months (mean)	Increase in size (≥20%)	28.4%
16	Ye LS	2020	410	Retrospective	≤20 mm SELs of second–fourth echo layer	EUS	28 months (median)	Increase in size, not specified	2.0%
17	Abe K	2022	824	Retrospective	≤20 mm SELs	EGD	5 years (median)	Increase in size (≥20%)	8.5%
18	Shiratori W	2023	145	Retrospective and prospective	GIMTs evaluated by EUS	EUS	5.2 years (mean)	Increase in size (≥25%)	26.9%
19	Choe Y	2023	135	Retrospective	SELs	EUS and EGD	52 mo (median)	Resection due to increase in size	14.8%
20	Iwamuro M	2024	560	Prospective	≤20 mm SELs	EGD	4.6 years (mean)	Increase in size (≥5 mm)	5.7%

NA, not available or not specified; SELs, subepithelial lesions; EUS, endoscopic ultrasonography; GIST, gastrointestinal stromal tumor; EGD, esophagogastroduodenoscopy; FNA, fine-needle aspiration; GIMTs, gastrointestinal mesenchymal tumors. * Includes SELs in organs other than the stomach. ** Analysis limited to doubling time only.

## Data Availability

No new data were created.

## Abbreviations

The following abbreviations are used in this manuscript:

SELs	Subepithelial lesions
GISTs	Gastrointestinal stromal tumors
EUS	Endoscopic ultrasound
